# Late-gadolinium enhancement predicts appropriate device therapies in nonischemic recipients of primary prevention implantable cardioverter-defibrillators

**DOI:** 10.1016/j.hrthm.2025.01.003

**Published:** 2025-01-10

**Authors:** Alan Kiang, Danah Al-Deiri, Tom Kai Ming Wang, Reza Nezafat, Diane Rizkallah, Thomas D. Callahan, Justin Z. Lee, Pasquale Santangeli, Oussama M. Wazni, Niraj Varma, Christopher Nguyen, Jakub Sroubek, Deborah Kwon

**Affiliations:** 1 Department of Cardiovascular Medicine, Section of Cardiac Electrophysiology and Pacing, Cleveland Clinic Foundation, Cleveland, Ohio; 2 Department of Cardiovascular Medicine, Section of Cardiovascular Imaging, Cleveland Clinic Foundation, Cleveland, Ohio; 3 Department of Radiology, Beth Israel Deaconess Medical Center, Boston, Massachusetts.

**Keywords:** Cardiovascular magnetic resonance, Implantable cardioverter-defibrillator, Nonischemic cardiomyopathy, Primary prevention, Sudden cardiac arrest, Ventricular tachycardia/fibrillation

## Abstract

**BACKGROUND:**

Better risk stratification is needed to evaluate patients with nonischemic cardiomyopathy (NICM) for prophylactic implantable cardioverter-defibrillators (ICDs). Growing evidence suggests that cardiac magnetic resonance (CMR) imaging may be useful in this regard.

**OBJECTIVE:**

We aimed to determine if late gadolinium enhancement (LGE) seen on CMR (dichotomized as none or minimal <2% vs significant ≥2%) predicts appropriate ICD therapies (primary endpoint) or all-cause mortality/transplant/left-ventricular assist device (LVAD) implantation (secondary endpoint) in patients with NICM.

**METHODS:**

We identified 344 patients with NICM who underwent primary prevention ICD implantation at Cleveland Clinic between 2003 and 2021 with CMR within 12 months before implant. LGE was calculated as percentage myocardium with pixel intensity ≥5 standard deviations higher than that of reference myocardium. Endpoints were adjudicated retrospectively by chart review.

**RESULTS:**

A total of 125 of 344 patients (36%) had none or minimal LGE, and 219 (64%) had significant LGE. Over a median follow-up of 61 months, 53 patients (24%) with significant LGE vs 10 (8%) with none or minimal LGE met the primary endpoint, and 56 patients (26%) vs 21 (17%) met the secondary endpoint, respectively. Significant LGE predicted the primary outcome in multivariable competing-risks regression (hazard ratio [HR] 2.99, 95% confidence interval [CI] 1.48–6.02, *P* = .002), but did not predict the secondary outcome in multivariable Cox regression (HR 1.34, 95% CI 0.78–2.29, *P* = .287).

**CONCLUSION:**

In patients with NICM and primary prevention ICDs, LGE ≥2% is predictive of appropriate device therapies but not all-cause mortality/LVAD/transplant. LGE may be a relatively specific predictor of sudden cardiac arrest risk and therefore could potentially be used during evaluation for prophylactic ICD implantation.

## Introduction

According to current guidelines, patients with nonischemic cardiomyopathy (NICM) are recommended to undergo implantation of a primary prevention implantable cardioverter-defibrillator (ICD) if left ventricular ejection fraction (LVEF) is less than or equal to 35% and New York Heart Association (NYHA) functional class is II or III despite guideline-directed medical therapy (GDMT).^1^ However, LVEF and NYHA class alone are relatively poor predictors of arrhythmic sudden cardiac arrest (SCA), as studies have demonstrated that the majority of patients receiving primary prevention ICDs by these criteria will never have an episode of sustained ventricular tachycardia (VT) or ventricular fibrillation (VF).^2^ Furthermore, large studies such as the DANISH trial (Danish Study to Assess the Efficacy of ICDs in Patients With Nonischemic Systolic Heart Failure) failed to show any benefit in all-cause mortality in such patients.^3^ Nonetheless, these patients are exposed to significant acute and long-term risks of an indwelling device. Therefore, better methods are needed to quantify the risk of SCA in NICM and improve patient selection for primary prevention ICD implantation.

Recently, there has been growing interest in correlating scar burden as measured by cardiac magnetic resonance (CMR) imaging with SCA risk. For example, in one of the largest studies to date, Leyva et al^4^ retrospectively examined 700 patients with NICM or ischemic cardiomyopathy (ICM) who underwent ICD, biventricular pacemaker (CRT-P), or biventricular defibrillator (CRT-D) implantation at a single center in the UK between 2002 and 2017 for both primary and secondary prevention indications. Although presence of late gadolinium enhancement (LGE) was associated with SCA, sustained VT/VF, or appropriate ICD therapy, absence of any LGE carried a remarkable negative predictive value of 99% for the same composite endpoint. More recently, similar findings were replicated by Hammersley et al^5^ in an even larger study that was limited to patients with NICM only.

The findings by Leyva et al and Hammersley et al are compelling and allude to the possibility of using CMR to screen for patients with a risk of SCA negligible enough to forego primary prevention ICD implantation even if they meet criteria for implantation under current guidelines. Additional data are needed to validate these findings and to better define the patient population that should potentially be targeted for CMR screening. Furthermore, subtle LGE is frequently identified in NICM, and it is unclear what amount of LGE is clinically significant and identifies NICM at risk for adverse outcomes. In this study we aimed to further validate the role of CMR in quantifying SCA risk, focusing specifically on patients with NICM undergoing primary prevention ICD implantation.

## Methods

### Study population

We performed retrospective chart review on all patients from 2003 to 2021 with NICM who received a primary prevention ICD at Cleveland Clinic and who also underwent CMR within 12 months before ICD implantation; CMR images had to be available for manual review for LGE quantification. The specific decision to pursue ICD implantation was based on the current clinical guidelines as well as the clinical judgment of the treating electrophysiologist. Patients undergoing cardiac resynchronization therapy (CRT) and those receiving a subcutaneous ICD (S-ICD) were included. Patients with a known diagnosis of specific high-risk syndromes (eg, amyloidosis, sarcoidosis, hypertrophic cardiomyopathy, arrhythmogenic right ventricular [RV] dysplasia) at the time of ICD implantation were excluded. ICD tachycardia detection programming followed institutional laboratory policy (2-zone, delayed detection programming, unless otherwise indicated). Baseline patient characteristics, including demographic information, past medical history, medical therapy and electrocardiographic (ECG) parameters were recorded. When available, preimplant 12-lead ECG tracings were manually reviewed and QT intervals were measured; the QTc interval was then calculated using Bazett’s formula.

### CMR

CMR studies were obtained on the following CMR platforms: 1.5-Tesla (Achieva, Philips Medical Systems, Best, the Netherlands; Sonata and Avanto, Siemens Medical Solutions, Erlangen, Germany) and 3-Tesla (Ingenia, Philips Medical Systems, Best, the Netherlands) machines. CVI-42 software (Circle Cardiovascular Imaging, Calgary, Alberta, Canada, version 5.17) was used for quantitative CMR analyses. Left and right ventricle quantification were performed on left ventricular (LV) short axis stack series using steady state free precession or other gradient echo sequences to quantify ejection fraction (LVEF and right ventricular [RVEF]). LV and RV dimensions were indexed to body surface area as follows: end- diastolic volume indexed (LVEDVi and RVEDVi), end-systolic volume indexed (LVESVi and RVESVi), stroke volume indexed (LVSVi and RVSVi), and left ventricular mass indexed (LVMi). Long- and short-axis LGE imaging was obtained 10 to 15 minutes after injection of 0.1 to 0.2 mmol/kg gadolinium dimeglumine. Quantification of LGE was performed on segmented inversion-recovery gradient echo sequences for studies performed in 2002 and 2003 and phase-sensitive inversion-recovery spoiled gradient echo sequences for studies performed after 2003. Epicardial and endocardial borders were segmented on the short axis LGE images, excluding the papillary muscles. A region of interest was manually drawn in an area of myocardium visually judged to be normal and without evidence of enhancement. LGE was quantified using the threshold definition of ≥5 standard deviation (SD) than user-defined viable myocardium, based on previous literature demonstrating 5 SD to be the threshold that best correlated with histopathology and visual assessment.^6–8^ LGE quantification was performed by a single blinded observer as outlined by the Society for Cardiovascular Magnetic Resonance (SCMR) guidelines^6^ and used the same technique as our previously published papers in our NICM cohort.^9,10^ Patients were dichotomized into no or minimal LGE burden (<2%) and significant LGE burden (≥2%), based on a previously determined LGE threshold of 2% from our previous work to identify association of LGE with higher risk of adverse outcomes in patients with NICM based on log-rank statistical analysis.^9^

### Clinical outcomes

Follow-up information was obtained through chart review. Only ICD interrogation reports and cardiology office notes were counted toward follow-up. At the 12-month mark following the last available ICD interrogation report or cardiology office note, patients who were not known to be deceased were censored as lost to follow-up even if they continued to have noncardiology encounters. The primary outcome was survival from appropriate ICD therapies including anti-tachycardia pacing (ATP) or shocks. The secondary outcome was survival from a composite of all-cause death, left ventricular assist device (LVAD) implantation, or heart transplant. The rate of inappropriate ICD shock therapies was also recorded. When available, all ICD interrogation information was manually reviewed by 2 investigators (A.K. and J.S.).

### Statistical analysis

Continuous variables were expressed as mean ± SD or median (interquartile range [IQR]) as appropriate. Categorical variables were expressed with absolute and relative frequencies. Differences in baseline characteristics were evaluated using the 2-tailed Student *t* test, Wilcoxon rank-sum test, or Fisher exact test, as appropriate. Survival probabilities were estimated using the Kaplan-Meier method, and the log- rank test was used to compare survival distributions between none or minimal and significant LGE groups. For the primary outcome, univariate and multivariate competing risks (Fine-Gray) regression models were used. For the secondary outcome, univariate and multivariate Cox proportional hazards regression models were used. Internal model validation was done using Harrell’s C-index, comparing the original dataset with a 200-samples bootstrap dataset. For both the primary and secondary outcome analyses, covariates with *P* < .05 in the univariate models were selected for inclusion in the multivariate models. Statistical analysis was performed using R version 4.3.2 (R Foundation for Statistical Computing, Vienna, Austria).

### Study approval

The study protocol was approved by the Cleveland Clinic Foundation Institutional Review Board.

## Results

### Study population

We identified a total of 372 patients meeting the inclusion criteria. However, CMR imaging data from 28 individuals were either fraught with poor quality or were unavailable for review; thus, the analysis was limited to 344 patients. Their baseline characteristics are shown in Table 1: Most patients were male (n = 210 [61%]), had a mean age of 57 ± 13 years and mean LVEF of 25 ± 9%. Notably, 44 (14.2%) patients did not meet the guideline-directed indication for primary prevention ICD implant: 21 (6.1%) had minimal symptoms (NYHA class I), and 33 (9.6%) had LVEF >35%. Subcutaneous ICDs were implanted in 31 (9.0%) patients, and a CRT system was used in 169 (49.1%) subjects.

**Figure 1:**
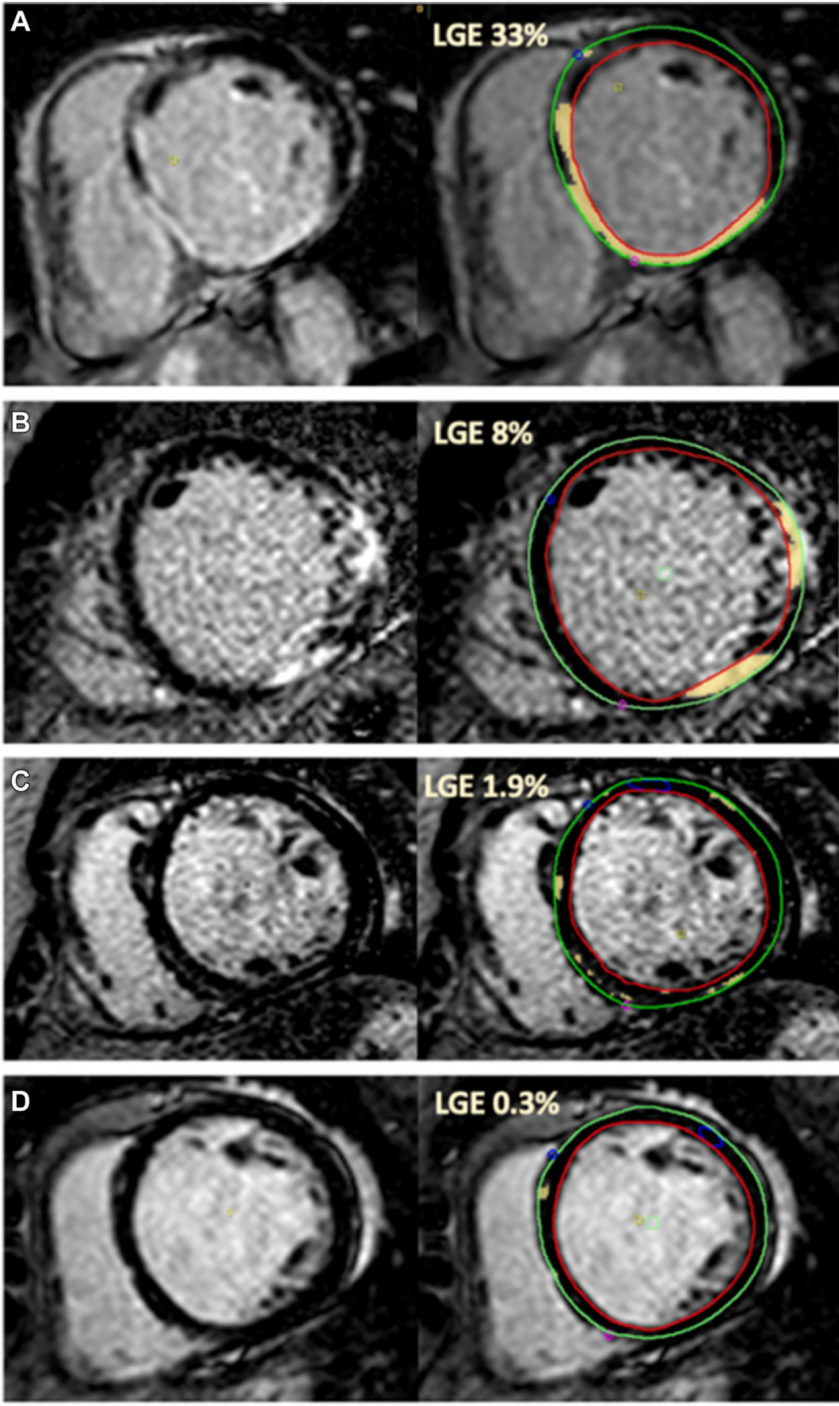
Representative MRI Images. Shown are representative MRI images of patients in this study. A and B: Patients with significant LGE burden who received ICD therapies. C and D: Patients with minimal LGE who received no ICD therapies. LGE % represents LGE burden for the entire left ventricle. Left ventricular walls are outlined with *green* and *red* contours; LGE distribution is highlighted in *yellow*. ICD = implantable cardioverter-defibrillator; LGE = late gadolinium enhancement; MRI = magnetic resonance imaging.

Review of CMR data identified 125 patients with no or minimal LGE (<2%), and 219 patients with significant LGE (≥2%). Differences in baseline characteristics that met statistical significance included a higher proportion of female patients (60 [48.0%] vs 74 [33.8%], *P* = .013) and a higher proportion of CRT recipients (77 [61.6%] vs 92 [42.0%], *P* = .001) in the no or minimal LGE vs significant LGE group. Mean RV end-diastolic volume index was also lower in the none or minimal LGE vs significant LGE group (82 vs 95, *P* = .006). Initial tachycardia-detection ICD programming was similar in both groups (minimum VT detection rate ≥180 beats per minute [bpm] was used in 120 [96.8%] of no or minimal LGE patients and 207 [94.1%] of significant LGE patients, *P* = .313). Representative CMR images are shown in Figure 1.

### Follow-up

**Figure 2:**
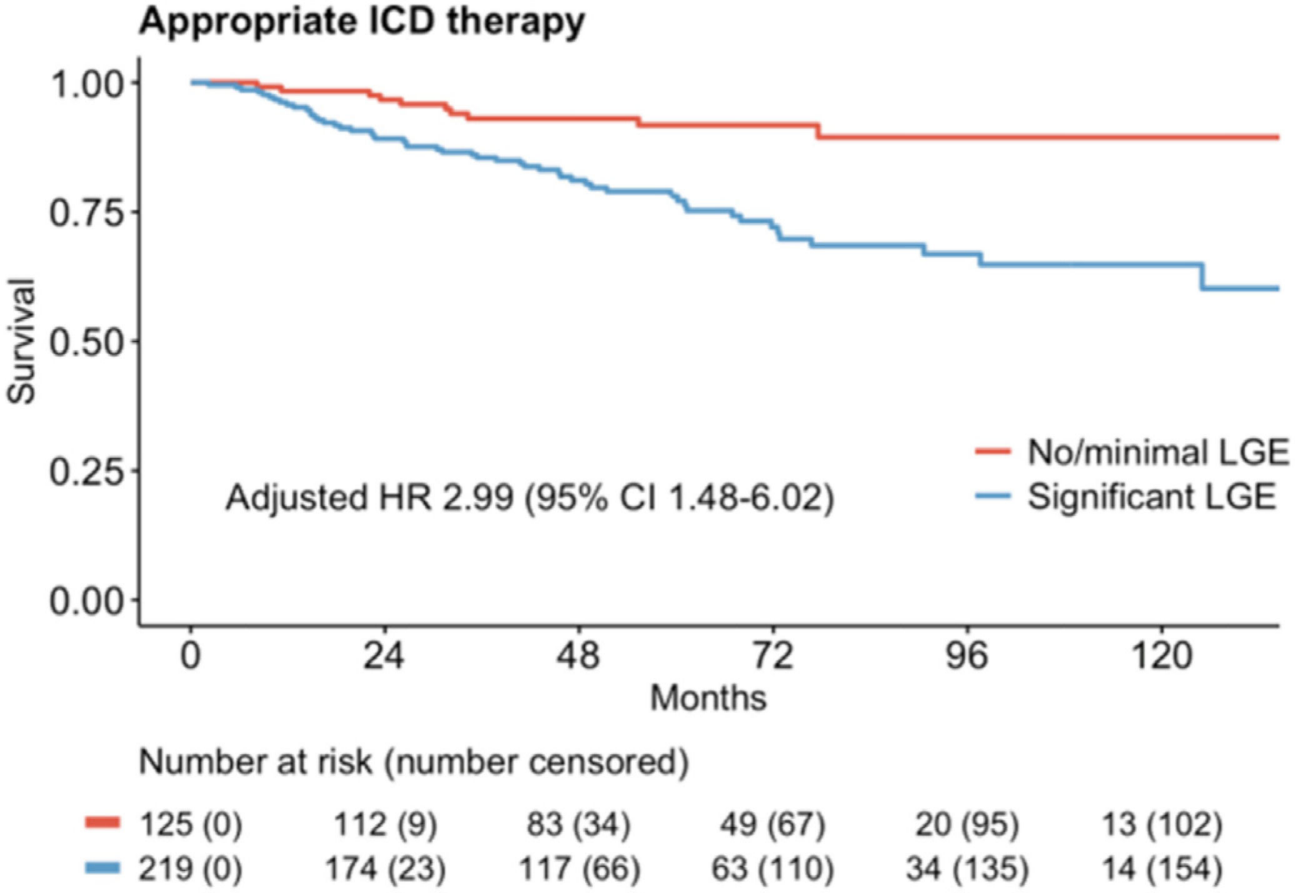
Primary outcome of appropriate ICD therapy in none or minimal vs significant LGE. CI = confidence interval; HR = hazard ratio; ICD = implantable cardioverter-defibrillator; LGE = late gadolinium enhancement.

Median follow up was 64.6 (IQR 44.7–87.1) months in the no or minimal LGE group and 59.3 (IQR 41.4–93.3) months in the significant LGE group. The primary outcome of appropriate ICD therapies occurred in 63 (18.3%) patients, and ICD interrogation data were available to the research team in 46 (73.0%) cases (Table 2). The primary event occurred in 10 of 125 patients (8.0%) in the none or minimal LGE group vs 53 out of 219 patients (24.2%) in the significant LGE group. This corresponded to a hazard ratio (HR) of 2.99 (95% confidence interval [CI] 1.48–6.02, *P* = .002; Figure 2) in an adjusted multivariable competing risk (Fine-Gray) regression model (Table 3). In the univariate model, significant LGE burden, absence of CRT, and use of any antiarrhythmic drug were significantly associated with the primary outcome (*P* < .05) and were therefore selected for inclusion in the multivariable model. Only LGE burden and CRT remained significant in the multivariable model. In the multivariable model, history of CRT resulted in a HR of 0.55 (95% CI 0.33–0.94, *P* = .027) for the primary outcome (Table 3).

**Figure 3:**
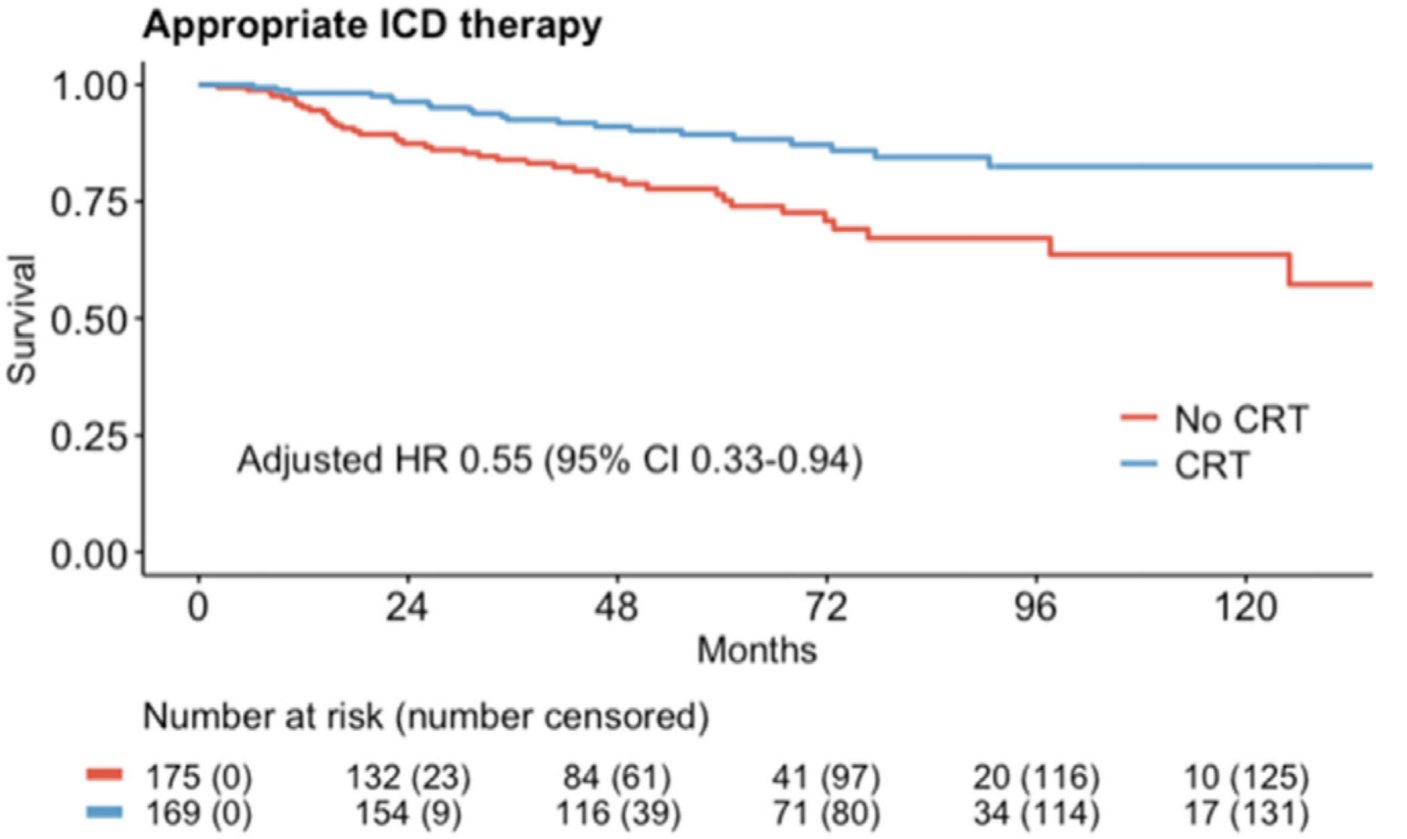
Primary outcome of appropriate ICD therapy in patients with CRT vs without CRT. CI = confidence interval; CRT = cardiac resynchronization therapy; HR = hazard ratio; ICD = implantable cardioverter-defibrillator; LGE = late gadolinium enhancement.

**Figure 4:**
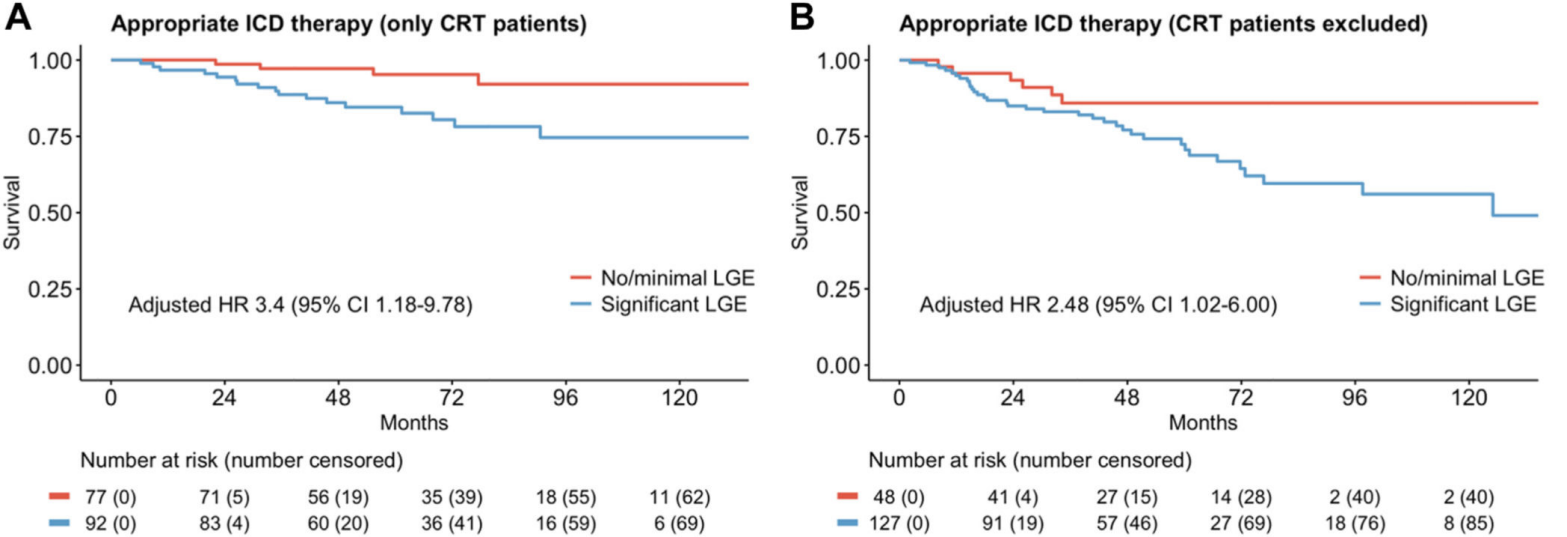
Primary outcome of appropriate ICD therapy in none or minimal vs significant LGE among (**A**) patients with CRT and (**B**) patients without CRT. CI = confidence interval; CRT = cardiac resynchronization therapy; HR = hazard ratio; ICD = implantable cardioverter-defibrillator; LGE = late gadolinium enhancement.

Comparison of the survival curves between patients with and without CRT showed that CRT was associated with a lower risk of ICD therapies (Figure 3). However, consistent with the multivariate regression model, LGE burden remained predictive of the primary outcome regardless of CRT status (Figure 4).

**Figure 5:**
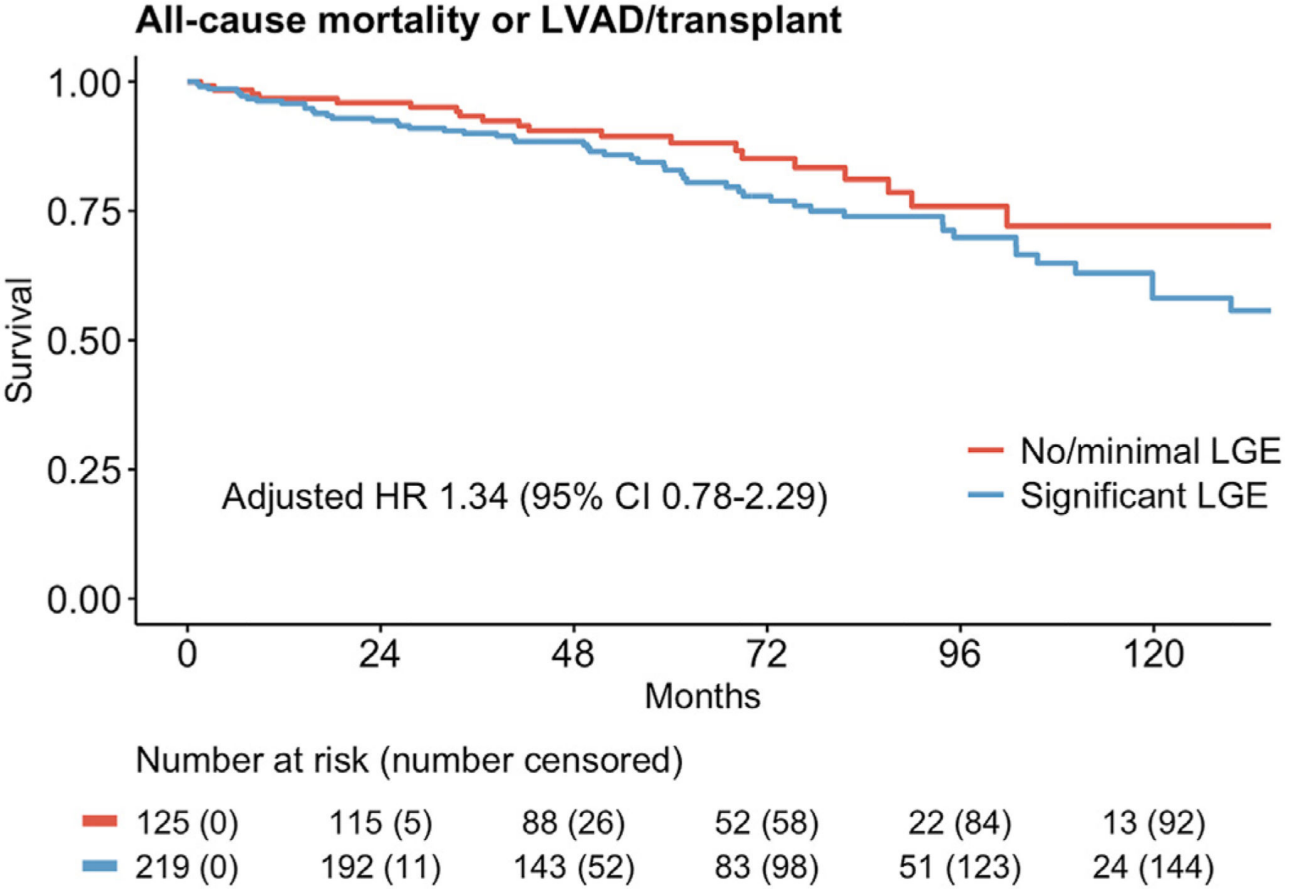
Secondary outcome of all-cause mortality/LVAD/transplant in none or minimal vs significant LGE. CI = confidence interval; HR = hazard ratio; LGE = late gadolinium enhancement; LVAD = left ventricular assist device.

The secondary outcome of all-cause death, LVAD implantation, or heart transplant was not significantly different between the none/minimal and significant LGE groups (Table 2 and Figure 5). Creatinine clearance (CrCl) >45 mL/min, presence of CRT, QTc interval, and beta-blocker use were significantly associated with the secondary outcome in univariate Cox regression (*P* < .05) and therefore selected for inclusion in the multivariate model, but only beta-blocker use, CrCl >45 mL/min, and CRT remained significant in multivariable analysis with HRs and 95% CIs of 0.12 (0.05–0.27, *P* < .001), 0.23 (0.12–0.44, *P* < .001), and 0.23 (0.13–0.42, *P* < .001), respectively (Table 4).

Internal validation of both models (primary and secondary endpoint analyses) using Harrell’s C-index method yielded consistent results (Supplemental Table S1 and Table S2). The models were also tested at different LGE burden cutoff points and receiver operating characteristic (ROC) curves were plotted (Supplemental Figure S1). In the primary endpoint analysis, the 2% LGE cutoff was near the ROC inflection point for optimal sensitivity.

**Figure 6:**
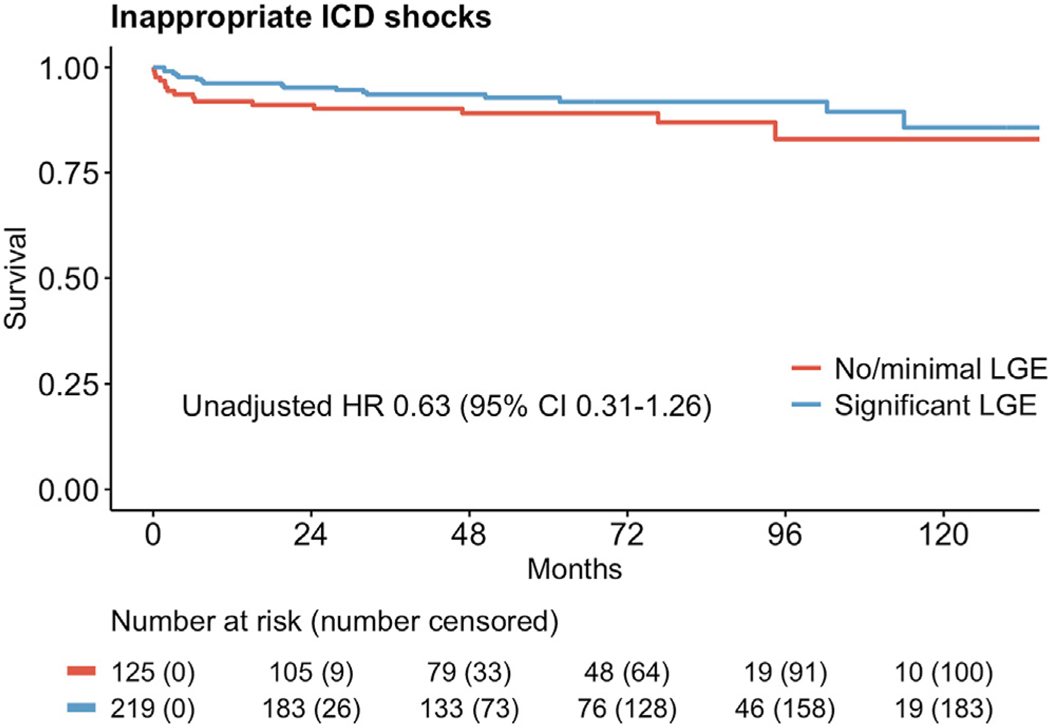
Inappropriate ICD shocks in patients with none or minimal vs significant LGE burden. CI = confidence interval; HR = hazard ratio; ICD = implantable cardioverter-defibrillator; LGE = late gadolinium enhancement.

Inappropriate ICD shocks occurred in 9.3% of patients overall (Table 2), and there was no significant difference be- tween none/minimal LGE and significant LGE groups (Figure 6). In multivariate Cox regression, atrial fibrillation was associated with a higher rate of inappropriate shocks, whereas CRT was associated with a lower rate, with HRs and 95% CIs of 2.98 (1.40–6.34, *P* = .005) and 0.44 (0.21–0.93, *P* = .032), respectively (Table 5).

### Appropriate ICD therapies in patients with none/minimal LGE burden

The clinical histories of the 10 patients in the none/minimal LGE group who received appropriate ICD therapies were examined (Supplemental Table S3). Two patients were later diagnosed with noncompaction cardiomyopathy and possible lamin A/C cardiomyopathy; both experienced sustained monomorphic VT that required ICD therapy. One patient required an ICD shock for torsade de pointes in the setting of markedly prolonged QTc (626 ms) caused by hydroxychloroquine therapy. One patient had frequent asymptomatic episodes of monomorphic nonsustained ventricular tachycardia (NSVT) at baseline but eventually received ATP in 1 instance, which resulted in a type-2 break shortly after the completion of a single round of ATP. The remaining 6 patients had no identifiable inherited cardiomyopathic diagnoses or reversible clinical triggers and presumably would have had a sustained arrhythmic event had they not received device therapy. Yet, even this small patient group was enriched for specific, potentially high-risk conditions (2 had severely reduced LVEF of ≤15%; 1 had end-stage renal disease treated with dialysis; 1 had pulmonary sarcoidosis, although without proven cardiac involvement; 1 had anthracycline-associated cardiomyopathy; and 1 had suspected alcohol-associated cardiomyopathy).

Conversely, 5 of the 10 treated patients with none or minimal LGE had LVEF of >35% at the time of ICD therapy (3 of whom were CRT recipients) and would thus not have qualified for de novo primary prevention ICD implantation per clinical guidelines.

## Discussion

In this retrospective observational cohort study of patients with idiopathic NICM who underwent primary prevention ICD implantation, we found that LGE burden (ie, ≥2%) is a significant independent predictor of appropriate ICD therapies, conferring an HR of 2.99 (95% CI 1.48–6.02, *P* = .002) in multivariable competing risk regression. Conversely, LGE burden was not a significant predictor of all-cause mortality, LVAD, or heart transplant in even univariate Cox regression. Together, these findings support the possibility of using LGE burden to identify patients with NICM who are at highest risk of arrhythmic SCA relative to the competing risk of all-cause death/LVAD/transplant, and therefore might benefit the most from a primary prevention ICD.

### Role of LGE in risk-stratification of patients with NICM

Risk stratification of patients with NICM has notoriously been a challenging feat. The relative lackluster nature of the data obtained by landmark trials may stem from the fact that these studies primarily relied on LVEF and NYHA functional class to guide patient selection.^3,11–13^ Both LVEF and NYHA class are nonspecific markers for mortality (including nonarrhythmic mortality), and their employment in risk stratification may potentially dampen the mortality effect ICDs may have. Conversely, the sensitivity of the LVEF/NYHA class-based risk-stratification model may also be limited. In fact, a large proportion of SCA events occurs in patients with relatively preserved LVEF (>35%), implying that ICDs may prevent some such adverse events if specific high- risk patients can be identified.^14,15^

Therefore, considerable effort has been directed toward identifying new, more specific SCA risk-stratification tools. Indeed, studies suggest that certain echocardiographic (such as LV sphericity) and electrophysiologic (such as QT-interval variability, microvolt T-wave alternans, and programmed ventricular stimulation) parameters may be useful in this regard.^14,16–18^

Furthermore, the N-terminal pro-B-type natriuretic peptide (NT-proBNP), a circulating marker of myocardial stretch, has been shown to convert from a nonspecific mortality marker to a specific predictor of appropriate ICD therapies once adjusted for renal function.^19^

Yet, perhaps the most encouraging body of evidence in this regard comes from studies of the prognostic power of CMRs. A landmark 2012 paper demonstrated that scar burden on CMR predicted a composite of death and ICD discharge in a mixture of ICM/NICM patients.^20^ A 2017 meta-analysis of 29 publications (2948 patients) exploring the role of CMR specifically in NICM substrate showed a strong association between presence of LGE and arrhythmic outcomes, regardless of LVEF.^21^ Moreover, the predictive ability of LGE was highest in studies focused on primary prevention ICD recipients (arrhythmic event annual rate of 17.2% vs 2.1%, *P* = .007, in individuals with and without LGE, respectively). Similar data were replicated in additional meta-analyses, including one published in 2024, again noting very low arrhythmic event rates in individuals without LGE.^22,23^ A 2019 propensity- matched analysis extended the role of CMR by observing that only patients with myocardial scar derived all-cause mortality benefit from ICDs.^24^ Indeed, some of the largest multi-center studies specifically designed to assess the prognostic utility of LGE in patients with NICM (although not always in the context of primary prevention ICD), identified myocardial fibrosis as a potent predictor of arrhythmic as well as nonarrhythmic outcomes.^25–27^ Conversely, a substudy of the DANISH trial identified the presence of LGE only as a nonspecific marker for all-cause mortality; accordingly, implantation of ICDs had no effect on outcomes of patient cohorts stratified by CMR findings.^28^

Many of these studies used a visual assessment of LGE as binary variable (presence vs absence). Therefore, the clinical significance and risk of subtle or minimal LGE is unknown. Our data employed a previously determined a quantitative threshold of LGE 2% in patients with NICM to predict all-cause mortality in a previous study.^9^ When using this LGE threshold in this NICM primary-prevention ICD cohort, our data provided additional insights regarding minimal LGE and mirror the bulk of the published evidence in favor of CMR findings as being a marker for arrhythmic outcomes; unlike some reports, our findings also highlight the specific nature of LGE as a predictor of appropriate ICD therapy rather than all-cause mortality. Indeed, almost 25% of patients with significant LGE received appropriate ICD therapies at some point during follow-up. In addition, by focusing specifically on the largest cohort to date of primary prevention ICDs in NICM, our work adds incremental value to the findings in the Leyva study, in which primary prevention NICM patients formed a relative minority.^4^ Additional work will be needed to further refine the specificity of CMR findings by incorporating LGE pattern and distribution into a risk-prediction algorithm.

On the other hand, the primary outcome of appropriate ICD therapies was still observed in 10 of 129 (8.0%) patients in the minimal LGE group (LGE <2%). If we were to exclude the 3 patients whose clinical histories later revealed either a high-risk or reversible condition (namely, the ones with LMNA cardiomyopathy, noncompaction cardiomyopathy, and acquired long QT), we are still left with an incidence of 5.6% (7 of 125 patients). This is still a considerable absolute risk that exceeds the published data.^4^ These findings can be explained through multiple potential mechanisms. First and foremost, appropriate ICD therapy is a sensitive but likely nonspecific surrogate of arrhythmic SCA, as not all treated arrhythmias would have resulted in SCA in the absence of ICD therapy. Second, cardiac remodeling may substantially alter the cardiac substrate, especially in studies with long follow-up times; in that sense, absence of LGE at an early timepoint may progressively lose its predictive value over time in some individuals. Third, although there is a clear mechanistic link between myocardial fibrosis and macroreentrant monomorphic VT, such association may be less strong with respect to polymorphic ventricular tachycardia (PMVT)/VF; indeed, 4 out of the 10 ICD-treated patients with none or minimal LGE burden received an ICD shock for PMVT/VF.

Our study also highlights the importance of weighing the risk of inappropriate ICD shocks in these patients. With nearly 1 in 10 study subjects experiencing an inappropriate ICD shock (independent of LGE status), the risk of such event is nontrivial and should be discussed extensively with any potential primary prevention ICD implant candidate.

### Additional risk factors

We observed that CRT was a protective factor with regards to appropriate ICD therapies, conferring an HR of 0.55 (95% CI 0.33–0.94, *P* = .027) in multivariable competing risks regression. The most intuitive explanation is that CRT indirectly protects against VT/VF by promoting recovery of LV function, which may be more feasible in patients without myocardial scar. However, the fact that the beneficial effect of CRT persists even after adjustment for LGE may indicate an additional, independent mechanism through which CRT protects again ventricular arrhythmias.^29^

Unlike LGE burden, the absence of CRT (along with reduced renal function and absence of beta-blocker therapy) was also predictive of all-cause death/LVAD/transplant, which may reflect the expected effect of CRT on cardiac mechanics and heart failure outcomes.

### Limitations

In addition to the limitations intrinsic to the single-center retrospective study design, this investigation may also be affected by a selection bias stemming from the fact that all study subjects underwent CMR imaging per the discretion of their treating clinician. In our chart review, we were unable to obtain data reliably on the rationale and indications for the CMRs that were obtained. However, a reasonable postulation is that a CMR is typically ordered if the treating clinician (typically a heart failure specialist) concludes that an infiltrative cardiomyopathy needs to be ruled out based on certain clinical, echocardiographic, or ECG features. For example, a patient with septal thinning on echocardiogram and conduction abnormalities or frequent ectopy on ECG might undergo a CMR to rule out cardiac sarcoidosis, whereas a patient with an unremarkable echocardiogram and ECG might be diagnosed with idiopathic NICM without undergoing CMR. It is possible that our study was enriched for patients like the former case and therefore could reflect a somewhat higher risk cohort compared with allcomers in NICM. With that in mind, one may be tempted to speculate that the selection bias in this study actually steered our results toward the null (ie, negative confounding). In other words, the predictive effect of LGE presence or absence could be even stronger if CMR data were available in all primary prevention ICD recipients with NICM. However, it should also be noted that the event rates in our study are comparable with the published data in trials with less presumed selection bias (for example, compared with the 18.3% rate of appropriate ICD therapies seen in our study, 17.4% and 11.5% ICD recipients in the DANISH trial experienced appropriate ATP and ICD shocks over a similar follow-up period, respectively).^3^

In a minority of patients enrolled in this study (14.2%), implantation of a primary prevention ICD was undertaken without a strong guideline-directed indication. This may limit the generalizability of the published findings. In addition, genetic testing was not uniformly performed in this population, so it is possible that individuals with certain high-risk genetic variants may have been inadvertently included in this study. Also, our LGE quantification was performed by a single observer; therefore, interobserver and intraobserver variability may be present and may limit our conclusions.

Heterogeneity in device programming could have also affected the incidence of both appropriate and inappropriate device therapies. That being said, we did make note of the lowest tachytherapy zone for all patients in our study and found that of 344 patients only 17 (4.9%) had their devices programmed to deliver therapies lower than 180 bpm at the time of implant.

Finally, the nonrandomized nature of this study resulted in an imbalance of baseline patient characteristics, most notably high use of CRT systems in patients with none or minimal LGE burden. Presence of this and other (possibly unidentified) confounders could affect the validity of our findings.

### Clinical implications and future directions

Our findings add to the growing body of research that specifically links the presence of myocardial scarring to clinical arrhythmic endpoints. This may amplify the importance of offering primary prevention ICD implantation to patients with concerning CMR findings, as long as they also have an existing guideline-directed indication for this intervention.

However, patients with LVEF ≤35% and NYHA class II-III but no evidence of LGE continue to pose a clinical challenge. Our data show that the rate of appropriate ICD therapies in this population (defined using a very conservative 2% LGE threshold) is very low and that most of these patients derive no benefit from ICDs. Yet, even in this population, the rate of potentially serious arrhythmic events is not completely negligible. Therefore, these patients should continue to be considered for ICD placement until evidence from a randomized clinical trial suggests otherwise. Additional advances in genetic screening and noninvasive electrographic monitoring may also be able to refine the identification of individuals at-risk for VT/VF mediated by a scar-independent mechanism.

As CMR imaging continues to be more widely available and affordable, further efforts (such as the ongoing GUIDEd study^30^) will also need to be directed to identifying patients with LVEF >35% who may derive benefit form ICDs.

## Conclusion

In patients with NICM who receive a primary prevention ICD, LGE burden assessed on CMR is strongly predictive of appropriate device therapies but not of all-cause mortality/LVAD/heart transplant, which suggests that it could potentially be used to identify patients within the NICM population who are specifically at risk of SCA and would benefit from prophylactic ICDs. Conversely, patients with minimal objective LGE burden derive little to no benefit from ICDs. This warrants further investigation with prospective cohort studies and randomized controlled trials.

## Supplementary Material

Supplementary Material

## Figures and Tables

**Table 1:** Baseline patient characteristics

		LGE burden		
	Overall (N = 344)	LGE – (LGE burden <2%) (N = 125)	LGE + (LGE burden ≥2%) (N = 219)	*P* value

Female gender, N (%)	134 (39.0)	60 (48.0)	74 (33.8)	.013
Age, years, mean (SD)	56.7 (13.2)	57.6 (12.6)	56.2 (13.5)	.350
Nonwhite ethnicity, N (%)	68 (19.8)	22 (17.6)	46 (21.0)	.534
BMI, kg/m^2^, mean (SD)	29.5 (6.4)	29.8 (7.1)	29.4 (5.9)	.594
History of diabetes, N (%)	64 (18.6)	24 (19.2)	40 (18.3)	.944
History of atrial fibrillation, N (%)	56 (16.3)	18 (14.4)	38 (17.4)	.575
Beta-blocker use, N (%)	329 (95.6)	118 (94.4)	211 (96.3)	.565
ACEi/ARB/ARNi use, N (%)	318 (92.4)	111 (88.8)	207 (94.5)	.086
Antiarrhythmic drug use, N (%)	32 (9.3)	9 (7.2)	23 (10.5)	.412
CrCl >45 mL/min, N (%)	319 (92.7)	114 (91.2)	205 (93.6)	.541
QTc, mean (SD)	440 (41)	443 (37)	438 (43)	.254
NYHA Class >II, N (%)	111 (32.3)	46 (36.8)	65 (29.7)	.215
CRT present, N (%)	169 (49.1)	77 (61.6)	92 (42.0)	.001
S-ICD present, N (%)	31 (9.0)	8 (6.4)	23 (10.5)	.279
LVEF, mean (SD)	25.0 (8.6)	25.0 (7.8)	26.0 (9.0)	.751
LVEDV index, mean (SD)	150 (47)	145 (45)	153 (48)	.113
LV mass index, mean (SD)	80 (23)	79 (23)	81 (22)	.513
RVEF, mean (SD)	39 (13)	41 (13)	38 (13)	.078
RVEDV index, mean (SD)	90 (32)	82 (28)	95 (33)	.006
Percent LGE, mean (SD)	4.3 (5.7)	0.7 (0.6)	6.4 (6.2)	<.001

ACEi = angiotensin converting enzyme inhibitor; ARB = angiotensin receptor blocker; ARNi = angiotensin-neprilysin inhibitor; BMI = body mass index; CrCl = creatinine clearance; CRT = cardiac resynchronization therapy; LGE = late gadolinium enhancement; LV = left ventricle; LVEF = left ventricular ejection fraction; LVEDV = left ventricular end-diastolic volume; RVEF = right ventricular ejection fraction; RVEDV = right ventricular end-diastolic volume; NYHA = New York Heart Association; SD = standard deviation; S-ICD = subcutaneous implantable cardioverter-defibrillator.

**Table 2:** Endpoints

		LGE burden		
	Overall (N = 344)	LGE – (LGE burden <2%) (N = 125)	LGE + (LGE burden ≥ 2%) (N = 219)	*P* value

Median follow-up, months (IQR)	61.0 [41.9, 90.8]	64.6 [44.7, 87.1]	59.3 [41.4, 93.3]	
Primary outcome: appropriate ATP or shock, N (%)	63 (18.3)	10 (8.0)	53 (24.2)	< .001
ATP only, N	29	5	24	
Shock, N	34	5	29	
Rhythm at primary event, N				
Monomorphic VT, N	43	6	38	
VF or polymorphic VT, N	20	4	15	
Secondary outcome: all-cause death,	77 (22.4)	21 (16.8)	56 (25.6)	.081
LVAD, transplant, N (%)				
Death, N	45	17	28	
Arrhythmic, N	1	1	0	
Cardiac, N	15	5	10	
Noncardiac, N	29	11	18	
LVAD, N	22	3	19	
Transplant, N	10	1	9	
Inappropriate ICD shock, N (%)	32 (9.3)	15 (12.0)	17 (7.8)	.268

ATP = anti-tachycardia pacing; ICD = implantable cardioverter-defibrillator; IQR = interquartile range; LGE = late gadolinium enhancement; LVAD = left ventricular assist device; VF = ventricular fibrillation; VT = ventricular tachycardia.

**Table 3:** Univariate and multivariate competing risks regression for the primary outcome of appropriate ICD therapies

	Unadjusted univariate model		Adjusted multivariate model	
Variable	HR (95% CI)	*P* value	HR (95% CI)	*P* value

Female gender	0.73 (0.43–1.24)	.240		
Age by decade	0.89 (0.74–1.07)	.230		
BMI	0.98 (0.94–1.02)	.290		
History of AF	1.25 (0.66–2.35)	.500		
Beta-blocker Use	1.47 (0.33–6.61)	.620		
CrCl >45 mL/min	0.68 (0.29–1.58)	.370		
LVEF	0.99 (0.96–1.02)	.410		
QTc interval	1.00 (0.99–1.00)	.130		
NYHA class >II	1.07 (0.64–1.79)	.810		
History of CRT	0.49 (0.29–0.82)	.006	0.55 (0.33–0.94)	.027
Antiarrhythmic drug use	2.06 (1.02–4.13)	.042	2.02 (0.99–4.15)	.055
Significant LGE (≥2%)	3.40 (1.72–6.72)	< .001	2.99 (1.48–6.02)	.002

AF = atrial fibrillation; BMI = body-mass index; CI = confidence interval; CrCl = creatinine clearance; CRT = cardiac resynchronization therapy; HR = hazard ratio; LGE = late gadolinium enhancement; LVEF = left ventricular ejection fraction; NYHA = New York Heart Association.

**Table 4:** Univariate and multivariate Cox regression for the secondary outcome of all-cause death/LVAD/transplant

	Unadjusted univariate model		Adjusted multivariate model	
Variable	HR (95% CI)	*P* value	HR (95% CI)	*P* value

Female gender	0.69 (0.43–1.13)	.138		
Age by decade	0.91 (0.77–1.07)	.245		
BMI	0.98 (0.95–1.02)	.284		
History of AF	1.51 (0.88–2.59)	.135		
Beta-blocker use	0.30 (0.15–0.63)	.001	0.12 (0.05–0.27)	< .001
CrCl >45 mL/min	0.33 (0.17–0.62)	.001	0.23 (0.12–0.44)	< .001
LVEF	0.98 (0.95–1.00)	.071		
QTc interval	0.99 (0.99–1.00)	.015	1.00 (0.99–1.00)	.283
NYHA class >II	1.46 (0.93–2.29)	.102		
History of CRT	0.27 (0.16–0.46)	< .001	0.23 (0.13–0.42)	< .001
Antiarrhythmic drug use	1.60 (0.85–3.04)	.148		
Significant LGE (≥2%)	1.55 (0.94–2.56)	.089	1.34 (0.78–2.29)	.287

AF = atrial fibrillation; BMI = body-mass index; CI = confidence interval; CrCl = creatinine clearance; CRT = cardiac resynchronization therapy; HR = hazard ratio; LGE = late gadolinium enhancement; LVAD = left ventricular assist device; LVEF = left ventricular ejection fraction; NYHA = New York Heart Association.

**Table 5:** Univariate and multivariate Cox regression for the secondary outcome of inappropriate ICD shocks

	Unadjusted univariate model		Adjusted multivariate model	
Variable	HR (95% CI)	*P* value	HR (95% CI)	*P* value

Female gender	0.96 (0.47–1.96)	.905		
Age by decade	1.07 (0.82–1.38)	.631		
BMI	1.01 (0.95–1.06)	.821		
History of AF	3.44 (1.68–7.03)	.001	2.98 (1.40–6.34)	.005
Beta-blocker use	1.27 (0.17–9.29)	.817		
CrCl >45 mL/min	0.92 (0.22–3.87)	.910		
LVEF	1.04 (1.00–1.08)	.041	1.02 (0.98–1.06)	.261
QTc interval	1.00 (0.99–1.01)	.631		
NYHA class >II	1.09 (0.53–2.27)	.810		
History of CRT	0.42 (0.20–0.90)	.025	0.44 (0.21–0.93)	.032
Antiarrhythmic drug use	1.41 (0.49–4.01)	.523		
Significant LGE (≥2%)	0.63 (0.31–1.26)	.192		

AF = atrial fibrillation; BMI = body-mass index; CI = confidence interval; CrCl = creatinine clearance; CRT = cardiac resynchronization therapy; HR = hazard ratio; LGE = late gadolinium enhancement; LVEF = left ventricular ejection fraction; NYHA = New York Heart Association.

## Abbreviations

ATP: antitachycardia pacing
CMR: cardiovascular magnetic resonance
CrCl: creatinine clearance
CRT-D/P: biventricular defibrillator/pacemaker
ICD: implantable cardioverter-defibrillator
ICM: ischemic cardiomyopathy
LGE: late gadolinium enhancement
LVAD: left ventricular assist device
LVEF/RVEF: left/right ventricular ejection fraction
NICM: nonischemic cardiomyopathy
NT-proBNP: N-terminal pro-B-type natriuretic peptide
NYHA: New York Heart Association
PMVT: polymorphic ventricular tachycardia
S-ICD: subcutaneous implantable cardioverter-defibrillator
SCA: sudden cardiac arrest
VF: ventricular fibrillation
VT: ventricular tachycardia
