# Supplementary material for: Late-gadolinium enhancement predicts appropriate device therapies in nonischemic recipients of primary prevention implantable cardioverter-defibrillators
Source: Heart Rhythm. Author manuscript; Available in PMC 2025 Jul 10. (PMC12241459; doi:10.1016/j.hrthm.2025.01.003)
Supplement: Supplementary Material [file NIHMS2070160-supplement-Supplementary_Material.docx]

**SUPPLEMENT**

Supplemental Table 1. Table showing the Harrell’s C-index (AUC) at three different time points for the multivariable Fine-Gray model for the primary endpoint on the original dataset, as well as the average C-index of the model when re-fitted on 200 bootstrapped training samples. There is little change in the C-index, suggesting that there is good internal validation of the model.

| **Dataset** | **Time (years)** | **Harrell’s C (AUC)** | **95% CI** |
| --- | --- | --- | --- |
| **Original Fine-Gray Model Dataset** | 1 | 68.9 | 53.8-84.0 |
|  | 5 | 69.7 | 62.4-76.9 |
|  | 10 | 66.3 | 56.4-76.3 |
| **Bootstrap (200 samples)** | 1 | 66.2 | 43.8-87.6 |
|  | 5 | 67.3 | 55.8-78.2 |
|  | 10 | 64.4 | 51.8-76.8 |

Supplemental Table 2. Table showing the Harrell’s C-index (AUC) at three different time points for the multivariable Cox model for the secondary endpoint on the original dataset, as well as the average C-index of the model when re-fitted to 200 bootstrapped training samples. There is little change in the C-index, suggesting that there is good internal validation of the model.

| **Dataset** | **Time (years)** | **Harrell’s C (AUC)** | **95% CI** |
| --- | --- | --- | --- |
| **Original Cox Model Dataset** | 1 | 79.7 | 66.7-92.8 |
|  | 5 | 71.0 | 62.9-79.1 |
|  | 10 | 67.0 | 55.5-78.6 |
| **Bootstrap (200 samples)** | 1 | 77.3 | 52.7-95.4 |
|  | 5 | 69.0 | 58.4-79.2 |
|  | 10 | 65.0 | 48.0-79.8 |

Supplemental Table 3. Clinical histories of patients with low LGE who received appropriate ICD therapies. Where available, ICD

interrogation strips documenting the first appropriate ICD therapy are shown.


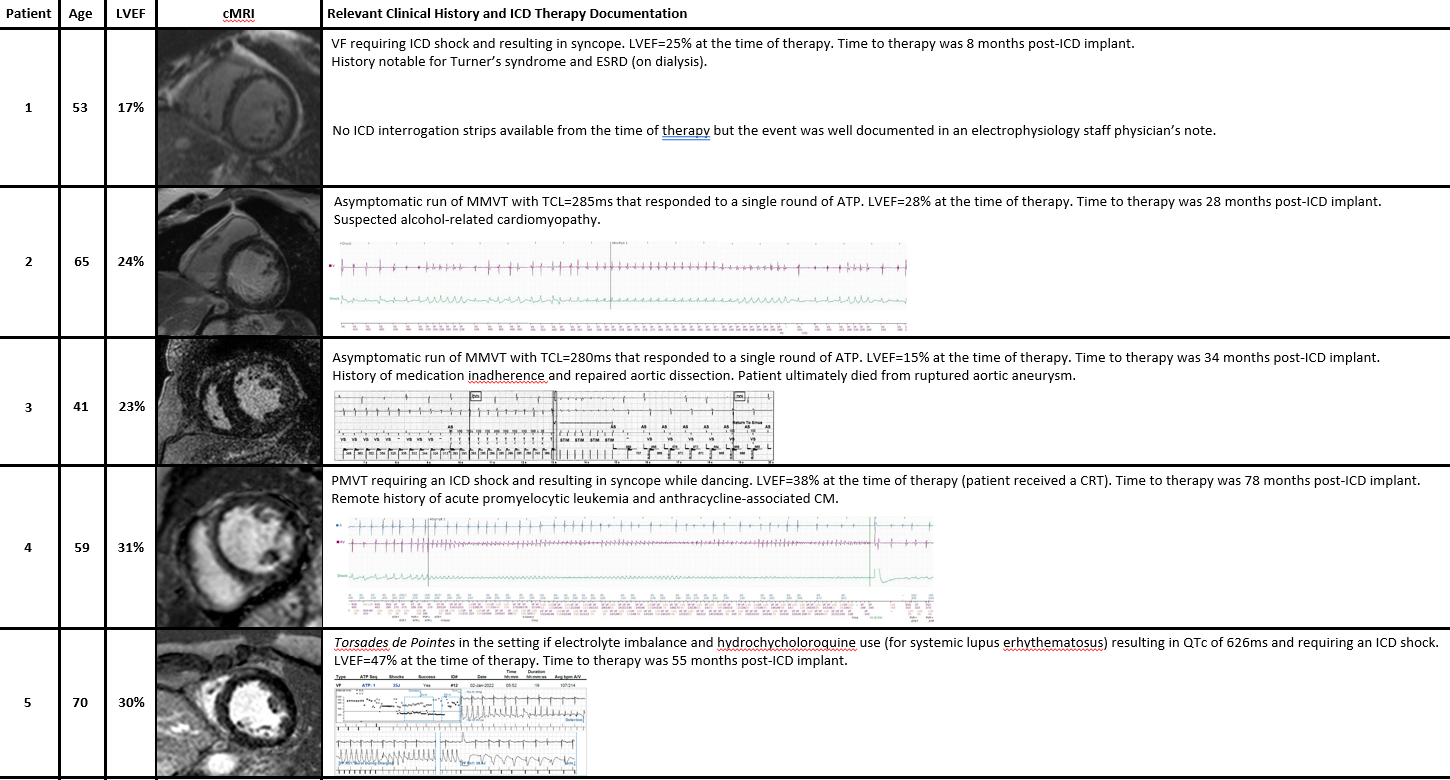

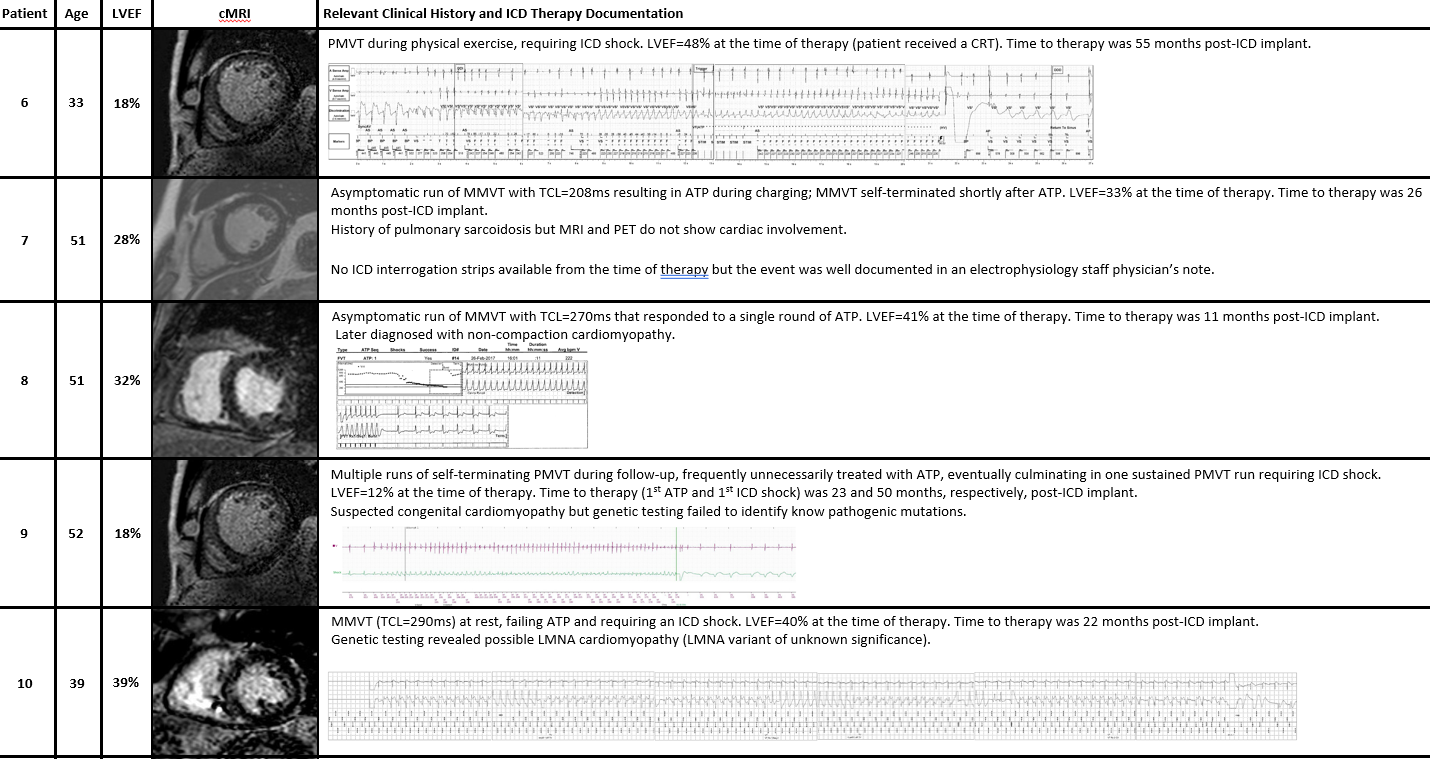


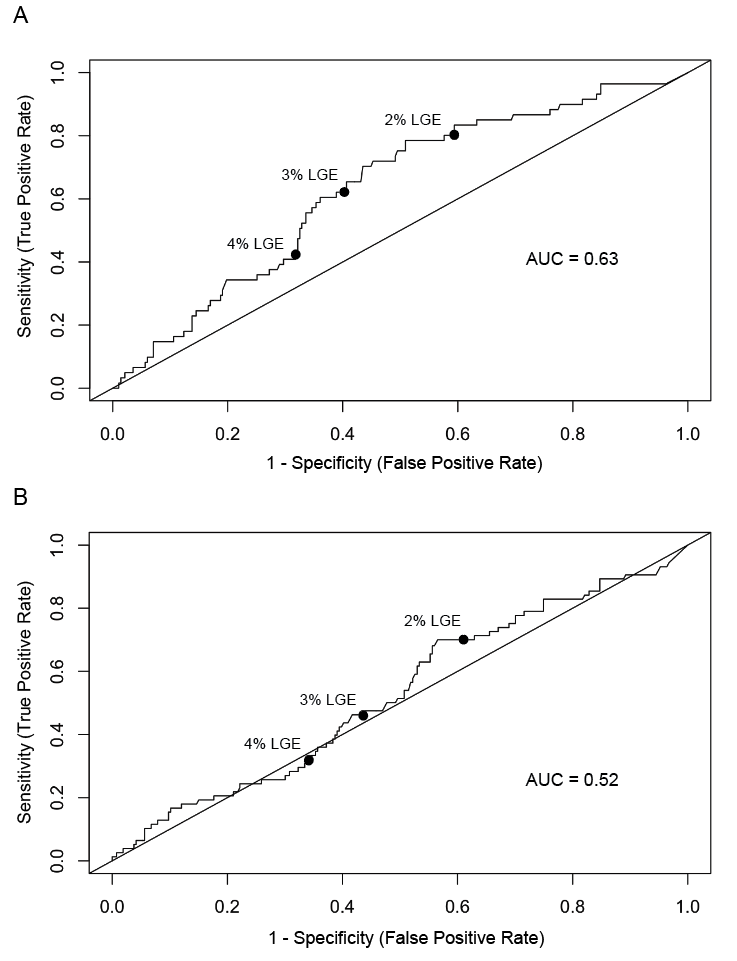


Supplemental Figure 1. Receiver operating characteristic (ROC) curves for LGE burden as a predictor of primary and secondary endpoints. A: ROC curve for the primary endpoint at 10 years using LGE 5SD. At 10 years, 61 out of 344 patients had met the primary endpoint, 256 had been censored, and 27 remained at-risk. B: ROC curve for the secondary endpoint at 10 years using LGE 5SD. At 10 years, 71 out of 344 patients had met the secondary endpoint, 236 had been censored, and 37 remained at risk.
